# Case Report: Double penile fracture

**DOI:** 10.12688/f1000research.16452.2

**Published:** 2019-08-21

**Authors:** Felipe Mercado-Olivares, J. Antonio Grandez-Urbina, Giomar Farfan-Daza, Juan Pacheco-Sauñe, Luciano Nuñez-Bragayrac

**Affiliations:** 1 Department of Medicine, Universidad San Martin de Porres, Lima, Peru; 2 Department of Urology, Clinica de Urologia Avanzada UROZEN, Lima, 15021, Peru; 3 Biomedical Research Institute, Universidad Ricardo Palma, Lima, Peru; 4 Universidad Continental, Lima, Peru; 5 Department of Urology, Hospital Nacional ESSALUD Alberto Sabogal Sologuren, Callao, Peru

**Keywords:** Penile Diseases, Urologic Surgical Procedures, Injuries, Penile induration

## Abstract

Penile fracture is an underreported surgical emergency. It usually occurs as a single rupture of the tunica albuginea in one of two corpora cavernosa; a rupture of both masses is an uncommon finding. We report a case of a young male who presented to the emergency department two hours after sustaining penile trauma. Prompt surgical exploration was performed four hours post-injury. He was found to have one fracture on each corpora cavernosa, without urethral injury, which were repaired successfully. The patient had a favorable recovery and was discharged on the third postoperative day without complications. The aim of this report is to highlight the importance of complete degloving of the penile shaft for a meticulous search during surgery to avoid missed injuries. This approach will ensure a successful outcome avoiding physical and psychological disabilities.

## Introduction

Fracture of the penis, or *faux pas du coit*, is an uncommon surgical emergency that occurs as a result of trauma to the erect penis ^1, 2^. The incidence of penile injuries is underreported because many patients do not seek medical attention ^3^. Penile fracture is defined as the rupture of the tunica albuginea and the corpora cavernosa ^4^. Most cases occur during sexual intercourse, usually due to hitting the symphysis pubis or the perineum after the penis slips out of the vagina; less commonly reported is during masturbation ^2, 3^.

It is manifested by a cracking or popping sound accompanied by immediate detumescence, followed by rapid swelling, widespread ecchymosis, sharp pain and deformity (away from the trauma site) ^2, 5^.

This condition can be quickly diagnosed after history taking, physical examination and imaging. Prompt diagnosis and early surgical repair are essential to ensure a successful outcome ^6, 7^.

It is uncommon for a penile fracture to involve both of the corpora cavernosa. We report a rare case of a double penile fracture and describe its presentation and management.

## Case report

**Figure 1.**
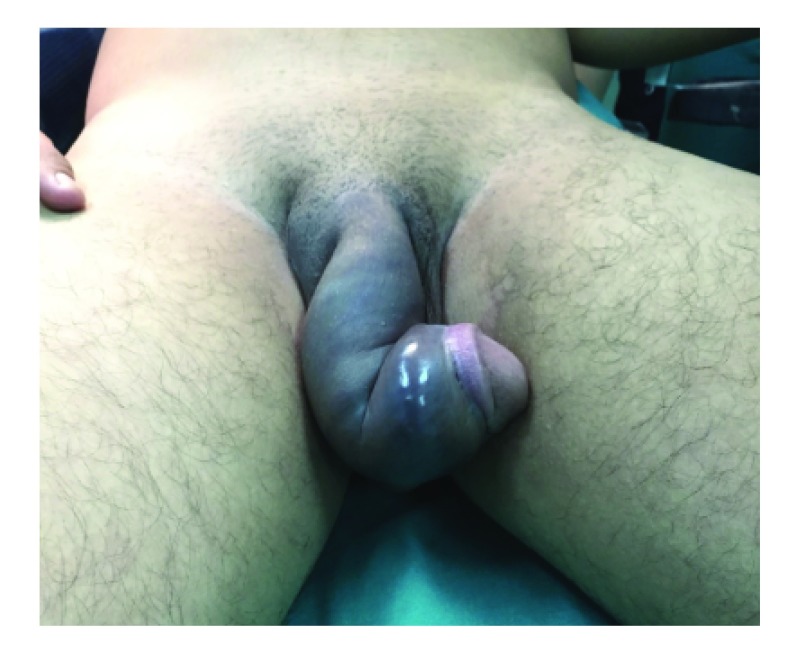
Swollen and deformed fractured penis.

A 32-year-old patient presented to the emergency department two hours after having penile trauma during vigorous sexual intercourse. He was having sexual intercourse in a 'woman-on-top' position when he heard a ‘snap’ sound followed by severe pain and immediate loss of tumescence. On physical examination, his penis was swollen, deformed, and with signs of ecchymosis. On admission, two ventral irregularities were found during penile palpation ( Figure 1). There was no urethral bleeding, nor voiding difficulties suggesting an uncompromised urethra. During the patient's consultation, no previous sexual disorders were found and the patient denied the usage of PDE5 inhibitors or intra-carvernosum injections. Labs results were within normal limits and radiological images were not obtained prior to surgical intervention due to lack of resources.

**Figure 2.**
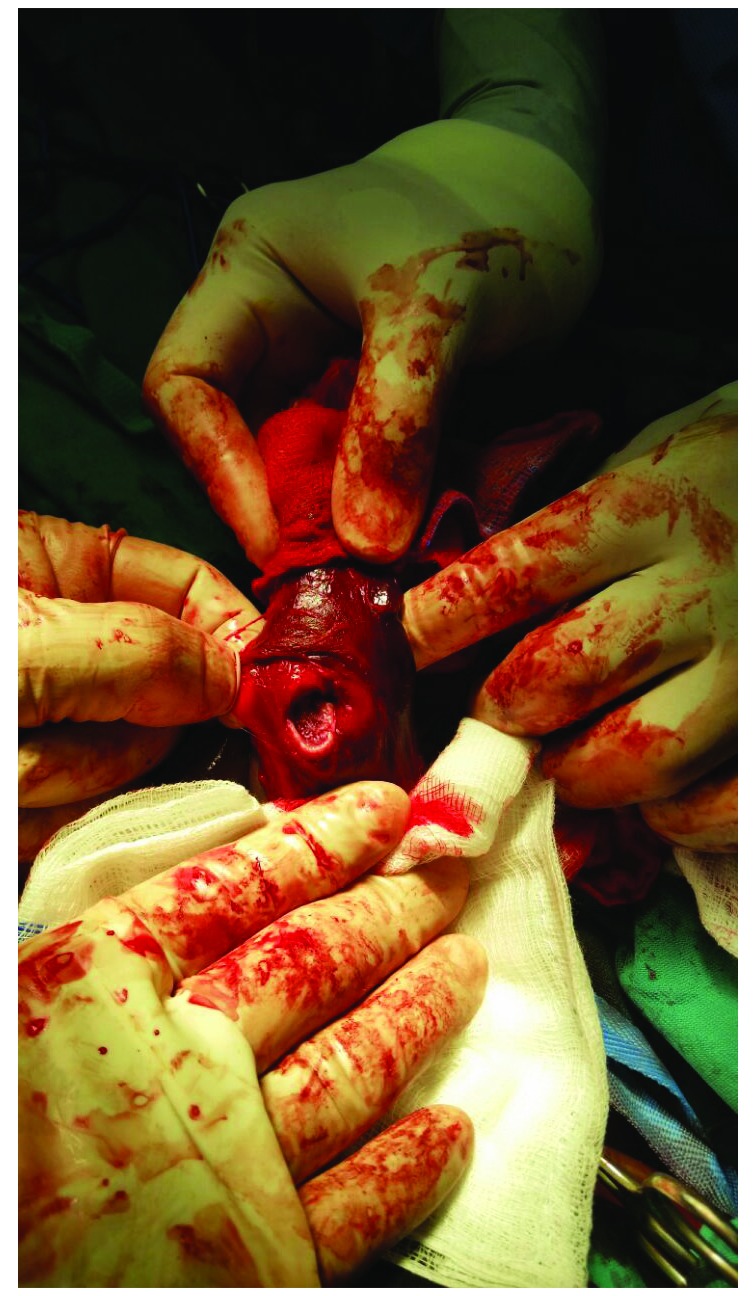
Exposure of one of the fractured corpora cavernosa after penile degloving during surgical treatment.

**Figure 3.**
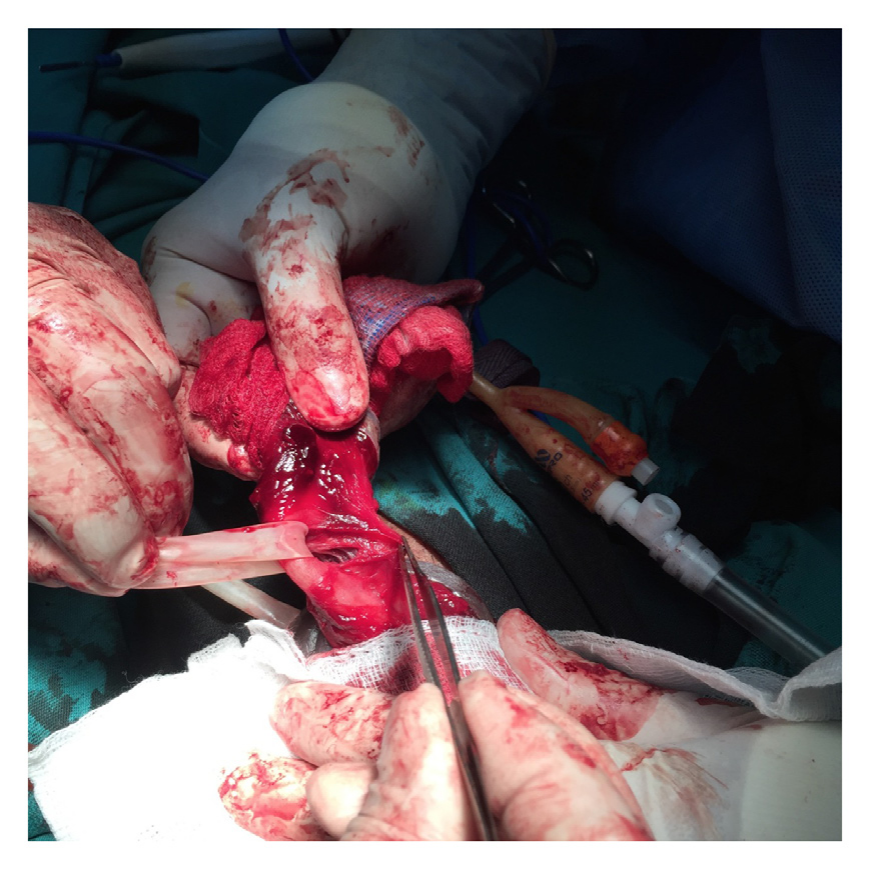
Protection of the urethra during surgery using a Penrose drain through the double fracture.

**Figure 4.**
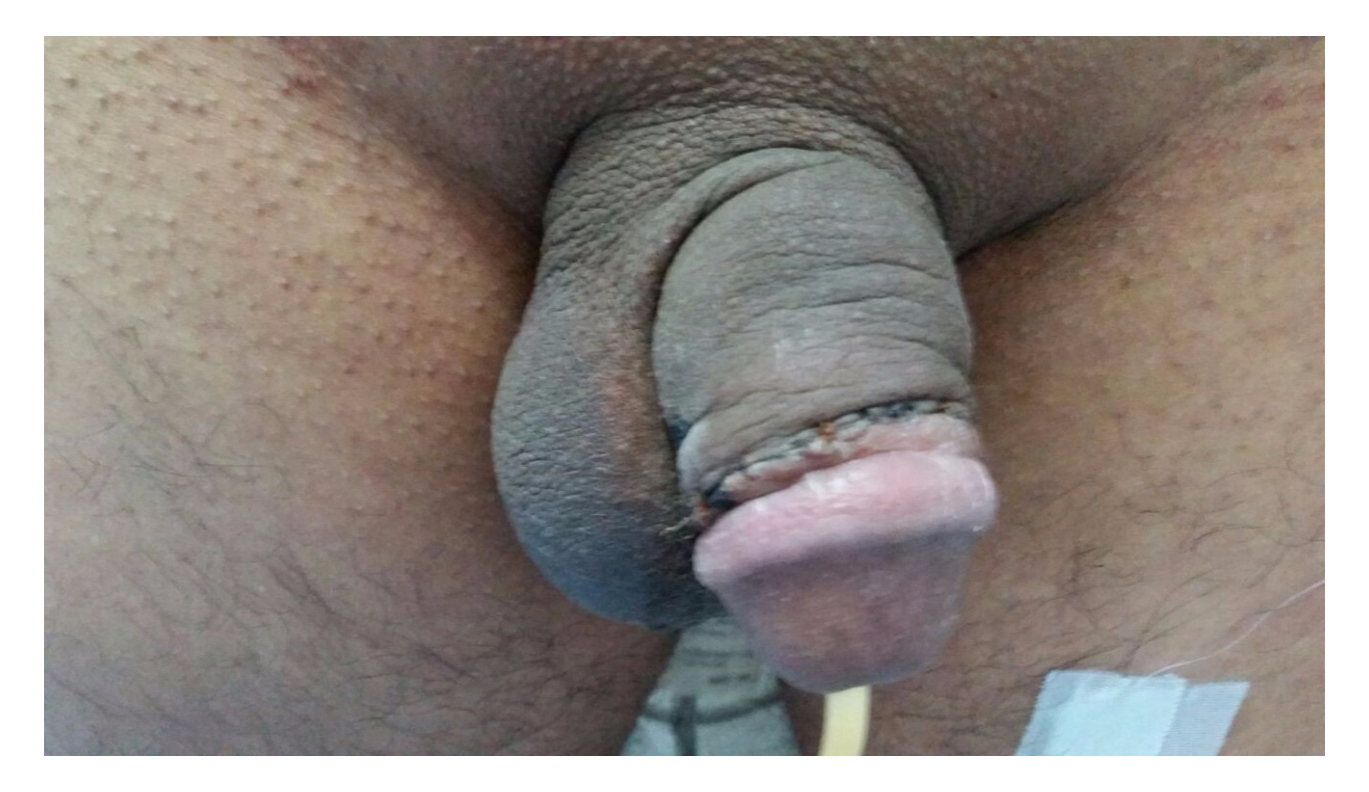
Second post-op day after repair of double penile fracture.

Cefazoline 2gr IV was administered prophylactically before surgery. Emergency exploration was performed 4 hours post-penile injury. A 16 Fr. Foley catheter was placed without difficulties. Then a circumcision was made, and surgical exploration was performed. On degloving the penile skin, a fracture on each corpora cavernosa was found. The length of the right and left defects were 25mm and 35mm, respectively ( Figure 2). In order to protect the urethra, a Penrose drain was used as a vessel loop through the double fracture separating it from the site of injury ( Figure 3). Repair of the two lacerations was done using 3-0 Vicryl in a continuous suture pattern. The Foley catheter was removed the next day without adverse events. The patient had a favorable recovery and was discharged three days after the surgical procedure without any complication ( Figure 4).

At a follow-up period of 90 days, the patient had a proper erectile function, no deformity, no pain during sexual intercourse, and no voiding difficulties.

## Discussion

The penis is composed of erectile tissue arranged in a columnar fashion. Two dorsolateral corpora cavernosa and one ventral corpus spongiosum, each enclosed by the tunica albuginea. The urethra traverse the corpus spongiosum throughout its length. The distal expansion of the corpus spongiosum forms the glans penis. Buck’s fascia encloses the corpus spongiosum ventrally, and splits dorsally to surround the two corpora cavernosa ^1, 4, 5^.

Penile fracture, or *faux pas du coit,* is an underreported but emergent urological condition ^8^. It’s underreported because many patients do not seek medical attention due to embarrassment, shame, or lack of guidance ^3^. Activities that can result in penile fracture includes self-manipulation to achieve tumescence, sexual intercourse, turning over in bed, a direct blow to the erect penis and interrupting the erection due to a violent bending of their penis called "Taqaandan" ^2, 4, 9^. The most common cause is sexual intercourse, with injuries often caused by different sexual positions. In an original article published in 2017, Barros *et al.* reported that ‘doggy style' was more commonly associated with double fractures out of 67 patients (10%) presented double corpus cavernosum lesión ^10^. In fact, any activity associated with tumescence can increase the chance of penile fractures. In 2002, Blake *et al.* reported the first case of a fracture related to pharmacologically induced erections ^11^. The increased risk of penile fractures during tumescence is related to the tunica albuginea stretching and thinning. The thickness of the tunica reduces from 2.4mm, in the flaccid state, to 0.25-0.5mm in the erect form 12.

The most extensive review of cases was made by Eke between 1935 and 2001. He analyzed 1331 cases from 183 publications. Most reports were from the Mediterranean region; the median age of patients were 35 years. Clinical features included sudden penile pain, detumescence, voiding difficulties, penile swelling and deviation. Associated injuries included urethral rupture. Complications of the rupture included coital difficulty, urethral fistula, penile plaque and erectile dysfunction ^1^.

The diagnosis is mainly clinical, although in the absence of typical signs it can pose a real challenge. Therefore, an adequate history and physical examination are cornerstones of the diagnostic process. In our patient, radiologic investigations were not performed due to lack of resources and the diagnosis was based solely on physical examination findings. Most cases reported in literature describes imaging modalities being used to exclude the presence of a concomitant urethral injury and to delineate the exact location of the albuginea rupture ^13^. Various imaging modalities have been used to aid in the diagnosis, such as cavernosography, retrograde urethrography, ultrasonography-colour Doppler, and magnetic resonance. Cavernosography is an invasive method that is rarely used. Retrograde urethrography is also an invasive method, but is required only if associated urethral injury is suspected ^7^. Ultrasonography is the modality most frequently used because of its cost and availability. Magnetic resonance imagine is the most accurate method to localize the lesions, but its availability is limited due to its cost ^14^. We would prefer to use MRI in this case in order to support the surgical decision, but the role of imaging is largely limited to unclear cases with equivocal findings or unreliable history ^15^.

Most studies report a solitary fracture of the corpora cavernosa. In our patient, both corpus were found to have fractures. Although no imaging modalities were performed, the omission of careful surgical exploration for the second site of injury might have led to unsatisfactory outcomes. Therefore, it seems essential to make a complete surgical exploration, degloving the penile shaft, while operating upon such cases.

Urgent surgery is the recognized gold standard approach. However, recent studies have revealed that long-term outcomes of early versus delayed repair in patients without urethral involvement are similar ^16, 17^. In 2011, Kozacioglu et al. evaluated 56 patients who underwent early and delayed penile fracture repair. Their study noted no serious deformities nor erectile dysfunction in the long term as a result of a delay in surgery in patients without urethral involvement ^5^. It would be useful to use intra-operative manoeuvres like urethral injection of methylene blue in order to detect occult urethral injuries. In our case, surgical repair was offered early in the course of the presentation. The outcome was favorable and no complications were noted on follow-up.

## Conclusion

Double rupture of the tunic albuginea is a rare finding in penile fractures. The diagnosis is clinical; however imaging should be used to help delineate the exact location and extent of injury. Degloving of the entire penis is recommended to identify possible multiple fractures, urethral damage, and damage to nearby structures. In our case, surgical repair was performed early which led to a successful recovery without complications. This case report is intended to further increase the literature helping physicians in search of experiences managing this low incidence condition.

## Consent

Written informed consent for publication of their clinical details and/or clinical images was obtained from the patient.

## Data availability

All data underlying the results are available as part of the article and no additional source data are required.
