# Supplementary material for: Case Report: Double penile fracture
Source: F1000Res. 2019 Aug 21;7:1828. Originally published 2018 Nov 21. [Version 2] doi: 10.12688/f1000research.16452.2 (PMC6733384; doi:10.12688/f1000research.16452.2)
Supplement: Peer review history

# Peer review: Reviewer response for version 2

**DOI:** 10.5256/f1000research.21347.r52773

**Published:** 2019-09-05

**Authors:** Sameer Trivedi

**Affiliations:** 1 Department of Urology, Institute of Medical Sciences, Banarus, India

The revised manuscript has addressed the concerns raised previously and appears suitable for indexing in it's present form.

I confirm that I have read this submission and believe that I have an appropriate level of expertise to confirm that it is of an acceptable scientific standard.

# Peer review: Reviewer response for version 1

**DOI:** 10.5256/f1000research.17983.r48955

**Published:** 2019-06-04

**Authors:** Sameer Trivedi

**Affiliations:** 1 Department of Urology, Institute of Medical Sciences, Banarus, India

1. The article has few grammatical errors and needs better proof reading.
2. Authors fail to mention other aetiological factors like the practice of “Taqaandan” in Middle Eastern countries.
3. Authors have stated that involvement of both cavernosa is uncommon in penile fractures but have failed to mention the incidence in absolute numbers.
4. The authors did not perform any radiological imaging study because of lack of resources. It implies that they would have preferred to perform some imaging procedure had the required resources been available. It would be interesting to know what imaging modality they would have preferred to use in such a clinical scenario.
5. The authors state that absence of urethral bleeding and voiding difficulties was an indicator of uncompromised urethra. However, it would be prudent to reiterate that both of these factors are not reliable in ruling out associated urethral injuries as an overlying clot can mask the urethral disruption. Even a retrograde urethrogram can be falsely normal in such settings. A high degree of suspicion, complete exposure of corpus spongiosum and use of intra-operative manoeuvres like urethral injection of methylene blue have been advocated to detect occult urethral injuries.
6. In discussion, authors have mentioned that there is no statistical difference in long-term outcomes of early versus delayed repair in patients without urethral involvement (Ref 14, 15). However, the quoted study by Naraynsingh et al (ref 14) is a case report, without any statistical analysis.
7. In conclusion, authors have stated that imaging should be used to delineate the location and extent of the injury. However, as per both AUA and EAU guidelines, role of imaging is largely limited to unclear cases with equivocal findings or unreliable history (Ref below in citations).

I confirm that I have read this submission and believe that I have an appropriate level of expertise to confirm that it is of an acceptable scientific standard, however I have significant reservations, as outlined above.

## References

[1] : European Association of Urology Guidelines on Urological Trauma. *European Association of Urology*.2018; Reference source

[2] : Urotrauma: AUA guideline. *J Urol*.2014;192(2) : 10.1016/j.juro.2014.05.004 327-35 10.1016/j.juro.2014.05.004 24857651PMC4104146

# Peer review: Reviewer response for version 1

**DOI:** 10.5256/f1000research.17983.r46660

**Published:** 2019-04-25

**Authors:** Rodrigo Barros

**Affiliations:** 1 Hospital Municipal Souza Aguiar, Rio de Janeiro, Brazil

This article describes a case of complex penile fracture (PF) with bilateral corpus cavernosum involvement resulting from sexual activity. Sexual intercourse represents the main cause of PF and is usually associated with high-energy traumas causing double fractures. This may occur during more vigorous sexual intercourse, especially in certain positions. According to our previous study, “doggy style” position was most frequently associated with complex PF, second only to the “man-on-top” position. Differently from this case report, in our sample, the “woman-on-top” position showed a low incidence, and no significant association with the severity of the PF. The authors should mention some aspects that can lead to complex bilateral lesions, such as vigorous sexual intercourse and certain types of sexual positions.

I confirm that I have read this submission and believe that I have an appropriate level of expertise to confirm that it is of an acceptable scientific standard.
